# High-dose-rate brachytherapy using molds for lip and oral cavity tumors

**DOI:** 10.1186/s13014-015-0390-z

**Published:** 2015-04-08

**Authors:** Teruhisa Unetsubo, Hidenobu Matsuzaki, Mitsuhiro Takemoto, Kuniaki Katsui, Marina Hara, Norihisa Katayama, Takahiro Waki, Susumu Kanazawa, Jun-ichi Asaumi

**Affiliations:** Department of Oral and Maxillofacial Radiology, Okayama University Graduate School of Medicine, Dentistry and Pharmaceutical Sciences, 5-1 Shikata-cho, 2-chome, Kita-ku, Okayama, 700-8525 Japan; Department of Oral Diagnosis and Dentomaxillofacial Radiology, Okayama University Hospital, 5-1 Shikata-cho, 2-chome, Kita-ku, Okayama, 700-8525 Japan; Department of Radiology, Japanese Red Cross Society Himeji Hospital, 12-1 Shimoteno, 1-chome, Himeji, 670-8540 Japan; Department of Radiology, Okayama University Hospital, 5-1 Shikata-cho, 2-chome, Okayama, 700-8525 Japan; Department of Radiology, Dentistry and Pharmaceutical Sciences, Okayama University Graduate School of Medicine, 5-1 Shikata-cho, 2-chome, Okayama, 700-8525 Japan

**Keywords:** Lip cancer, Oral cavity cancer, Mold, High-dose-rate, Brachytherapy

## Abstract

**Background:**

High-dose-rate (HDR) brachytherapy using the mold technique is a less invasive treatment for early lip and oral cavity cancer. However, limited reports exist regarding the feasibility of this method. In this retrospective study, we evaluated the outcome of this therapy and investigated its feasibility for lip and oral cavity tumors.

**Methods:**

Between May 2002 and December 2010, 17 patients (median age, 80.0 years) with histologically confirmed squamous cell carcinoma of the lip or oral cavity were treated by means of HDR brachytherapy using the mold technique after external beam radiotherapy (EBRT). Tumor sites included the buccal mucosa in eight cases, the gingiva in three cases, the lips in two cases, the floor of the mouth in two cases, and the hard palate in two cases. For all patients, EBRT (30 Gy/15 fractions), was performed before HDR brachytherapy. Two 6-Gy fractions were delivered twice daily for 2 days a week with an interval of 6 hours between the fractions. The total HDR brachytherapy dose was 24 Gy. Prior to EBRT, two patients with neck metastasis underwent neck dissection, and one patient with an exophytic tumor underwent tumor resection.

**Results:**

The median follow-up period was 53.4 (range, 4.8–83.4) months. Of the 17 patients, 14 (82.4%) achieved a complete response, and three (17.6%) displayed a partial response.

The overall 3- and 5-year survival rates were both 68.8%, the 3- and 5-year disease-specific survival rates were both 86.7%, and the 3- and 5-year local control rates were both 54.1%. Seven patients developed local recurrence at a median time of 3.4 (range, 1.7–29.1) months after treatment. Nodal and lung metastases occurred separately in two patients. By the end of the follow-up period, two patients had died of the primary disease and four patients had died of other causes.

**Conclusions:**

Although there is a need to improve the technical aspects of the treatment protocol, HDR brachytherapy using the mold technique might be a therapeutic option for superficial lip or oral cavity tumors, especially in older patients who have a poor performance status or are in poor physical condition.

## Background

Low-dose-rate (LDR) and high-dose-rate (HDR) brachytherapy are often employed for the radical treatment of early-stage lip and oral cavity cancer, because they preserve the shape and function of tissues and organs to a greater degree than surgical treatment [1,2]. LDR and HDR brachytherapy achieve equivalent therapeutic outcomes, but LDR brachytherapy requires the patient to be isolated [1,2]. Khalilur et al. reported that LDR brachytherapy was safe for very elderly patients with tongue carcinoma and achieved outcomes comparable with those seen in younger patients [3]. However, it is sometimes difficult to treat patients who have a poor performance status or a declining physical condition with LDR brachytherapy because of the need to isolate them.

Remote afterloading HDR brachytherapy using an Ir-192 microsource has resolved this problem, but this technique is mainly used for interstitial irradiation of carcinomas of the lip, tongue, and floor of the mouth [1,2,4-6]. Although intracavitary irradiation is a less invasive treatment than interstitial irradiation, it is rarely used for oral cavity cancers. The limited use of intracavitary irradiation is attributable to the limited treatment provided for superficial oral tumors, and the absence of oral equivalents of the off-the-shelf applicators used to treat cancer of the uterine cervix, such as the tandem and ovoid applicator. For superficial oral cavity tumors, some authors (including us) have reported the effectiveness of HDR brachytherapy using a mold [7-12]. However, these previous studies only included a few cases with short follow-up periods [7-12]. The aim of this retrospective study was to evaluate the therapeutic outcome of this method and investigate its feasibility for lip and oral cavity tumors.

## Methods

### Patients

Between May 2002 and December 2010, a total of 17 patients (10 males and 7 females) with lip or oral cavity tumors were treated using mold therapy with an HDR remote afterloading unit and an Ir-192 microsource (microSelectron HDR: Nucletron Co., Veenendaal, The Netherlands) at Okayama University Hospital. All tumors were histologically identified as squamous cell carcinoma. The characteristics of the patients are shown in Table 1. Their median age was 80 (range, 59–94) years (mean age, 78.2 years).

**Table 1:** **Baseline Characteristic of patients (n = 17)**

**Characteristic**	**Values**
Age (years)	
Median	80.0
Range	59-94
≤ 70	3
> 70	14
Gender	
Male	10(58.8%)
Female	7(41.2%)
ECOG Performance status	
0	4(23.5%)
1	9(53.0%)
2	4(23.5%)
Tumor site	
Buccal mucosa	8(47.1%)
Gingiva	3(17.6%)
Lip	2(11.8%)
Floor of the mouth	2(11.8%)
Hard palate	2(11.8%)
T stage	
T1	1(5.9%)
T2	11(64.7%)
T3	5(29.4%)
N stage	
N0	15(88.2%)
N1	1(5.9%)
N2b	1(5.9%)
Stage	
I	1(5.9%)
II	9(52.9%)
III	6(35.3%)
IVA	1(5.9%)

Of the 17 patients, 15 had primary tumors and two had recurrent tumors. Four, nine, and four patients displayed Eastern Cooperative Oncology Group performance statuses of PS0, 1, and 2, respectively. The tumor sites were as follows: buccal mucosa, eight cases; gingiva, three cases; lip, two cases; floor of the mouth, two cases; and hard palate, two cases. In accordance with the 2009 Union for International Cancer Control system, the tumor stage was T1 in one patient, T2 in 11 patients, and T3 in five patients. Two patients had regional lymph node metastasis (N1 and N2b), three patients with PS0 refused surgical treatment, and 14 patients could not undergo radical surgical treatment for the primary tumor because of their age, performance status, poor physical condition, and/or family problems. In addition, it was difficult for patients to undergo interstitial LDR brachytherapy because of the need to isolate them. When determining the indications for HDR brachytherapy, we expected that mold therapy could be used as a radical treatment for cases such as some buccal mucosa T1, hard palate and gingiva. However, we treated patients using mold therapy as the second choice, even in cases such as some buccal mucosas at stages >T2, and others involving the adaptation region such as the floor of the mouth; this was because there was no choice but to use conventionally-fractionated external beam radiation, and we could not choose a palliative irradiation. All patients provided written informed consent. The research protocols for this study were approved by the ethical committee of Okayama University Graduate School of Medicine, Dentistry, and Pharmaceutical Sciences (Number: 1860).

### Radiotherapy

Two patients with lymph node metastasis underwent upper neck dissection before starting radiotherapy. One patient with an exophytic tumor in the buccal mucosa underwent partial tumor resection to decrease the thickness of the tumor prior to the commencement of radiotherapy.

**Figure 1:**
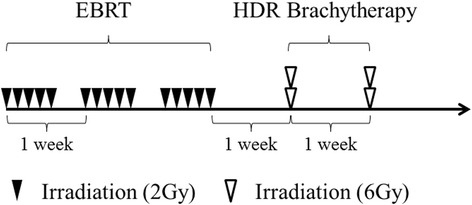
**Radiotherapy treatment schedule.** EBRT: External beam radiotherapy, HDR: High-dose-rate.

**Figure 2:**
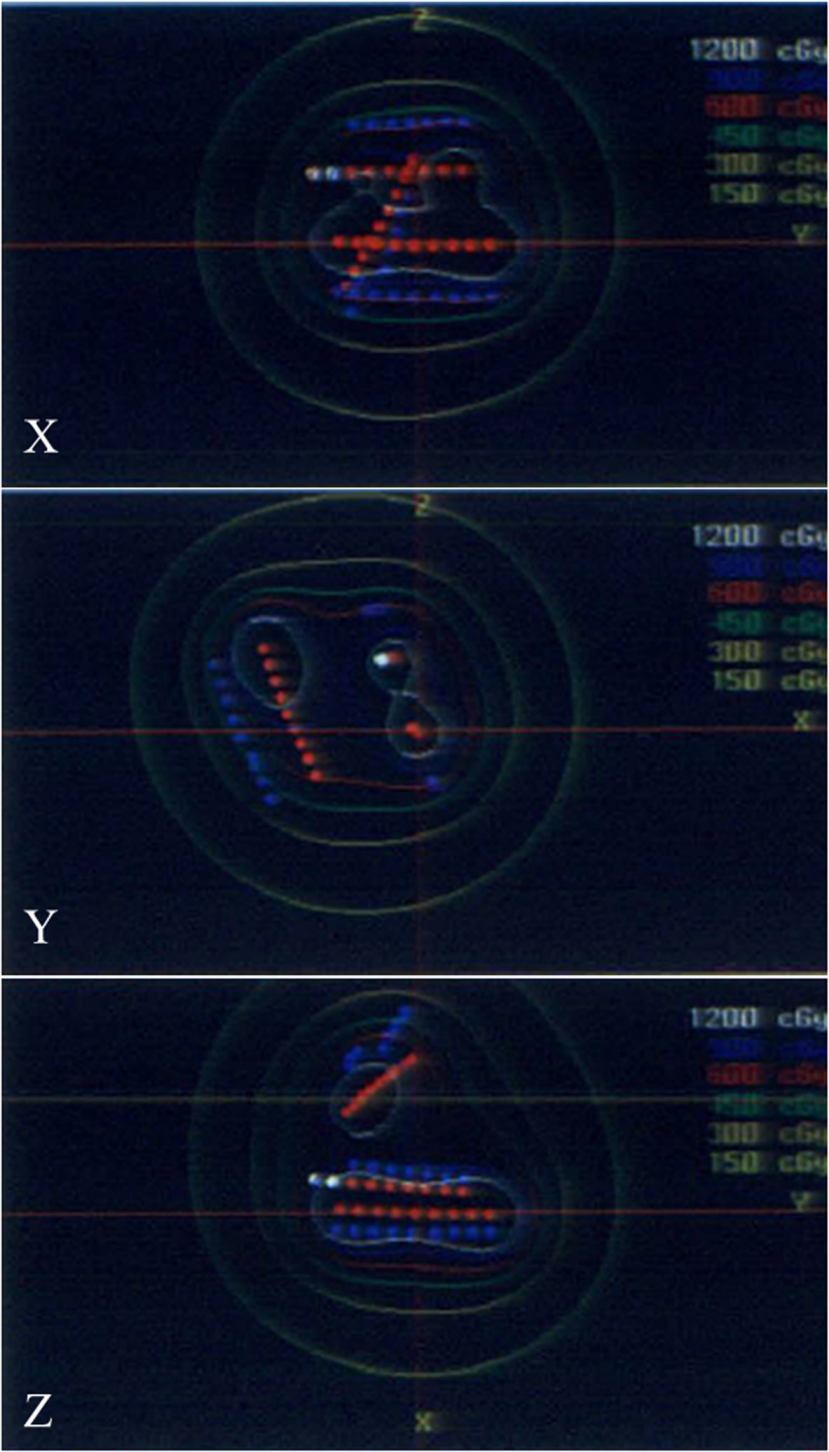
**Dose distribution of RALS.**

The radiotherapy treatment schedule is detailed in Figure 1. In all patients, external beam radiotherapy (EBRT) involving a radiation dose of 30 Gy (2 Gy/day, five fractions/week) was performed with 4-MV X-rays before HDR brachytherapy. All patients were treated with parallel opposing lateral or anterior-posterior fields. Before HDR brachytherapy, a mold made of resin was prepared for each patient. Prior to embedment of the catheters into the mold, the gross tumor volume (GTV) was determined after EBRT. We defined the area displaying mucositis after EBRT as the GTV by direct inspection and using findings from magnetic resonance imaging and/or ultrasonography performed before EBRT. To minimize the area at risk of complications, we used the GTV as the clinical target volume (CTV), and 5 mm was added to the CTV to obtain the planning target volume (PTV). A median of 4 (range, 3–10) catheters were embedded in the mold at parallel intervals of 10 mm so that they could cover a sufficient proportion of the PTV. For six patients we used the two-piece mold technique, which is a method for shifting the irradiation plane by dividing the mold for the tumors close to the commissure of the lips [12]. Because the catheter was hard and had poor flexibility, an adequate radiation dose could not be administered to a tumor that had extended towards the skin region from the corner of the mouth, or near the corner of the mouth. Indeed, it is not the way that the tumor is irradiated from the skin side. The dose reference point was set at 5 mm below the surface of the mucosa for all patients. The dose distributions were calculated using a computer software program (Plato Brachytherapy: Nucletron Co., Veenendaal, The Netherlands). HDR brachytherapy was performed after EBRT. The interval between EBRT and HDR brachytherapy had a median duration of 8 (range, 7–20) days. Two 6-Gy fractions were delivered twice a day for 2 days a week with an interval of 6 hours between the fractions. The total HDR brachytherapy dose was 24 Gy (Figure 2).

Overall survival, disease-specific survival (DSS), and local control rates were calculated from the date of the first treatment to the events of interest. The local control rates were determined from imaging findings, clinical physical examinations, or pathological tissue findings. All survival data and local control rates were estimated according to the Kaplan-Meier method.

All symptoms were classified according to the Common Terminology Criteria for Adverse Events version 4.0 when applicable.

## Results

Treatment was not suspended due to radiation-induced complications or other reasons in any patient. In one patient with buccal cancer, EBRT did not result in sufficient tumor shrinkage. Hence, part of the tumor was resected to decrease its thickness before HDR brachytherapy was started, and the interval between EBRT and HDR brachytherapy in this patient was 20 days.

The median total treatment period was 38 (range, 31–49) days. The radiation source strength for each HDR brachytherapy fraction ranged from 161.7–349.2 GBq. All patients experienced grade 1 (nine patients) or 2 (eight patients) acute mucositis. The six patients treated with two-piece molds suffered from grade 2 dermatitis. After HDR brachytherapy, a complete response (CR) was achieved in 14 patients (82.4%), and a partial response (PR) was achieved in 3 patients (17.6%). Of the three patients who achieved a PR, two had tumors in the buccal mucosa and one had a tumor in the floor of the mouth. One patient (buccal mucosa) received additional HDR brachytherapy (24 Gy in 4 fractions over 2 weeks) 4 months after the initial treatment. One patient (floor of the mouth) underwent tumor resection and remained disease-free until the end of the follow-up period. The remaining patient (buccal mucosa) underwent stereotactic radiotherapy (SRT) with a CyberKnife (Accuray Inc., Sunnyvale, CA, USA) at a total dose of 35 Gy delivered in 5 fractions.

**Figure 3:**
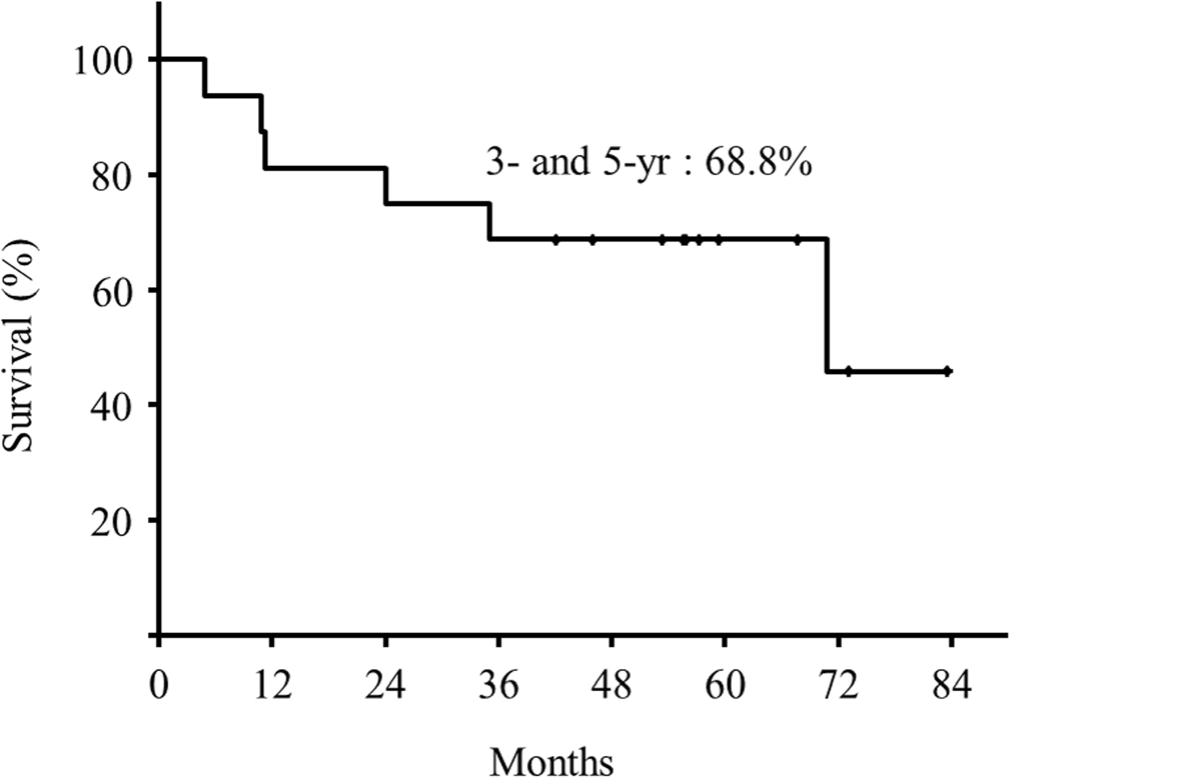
**Overall survival rate.**

**Figure 4:**
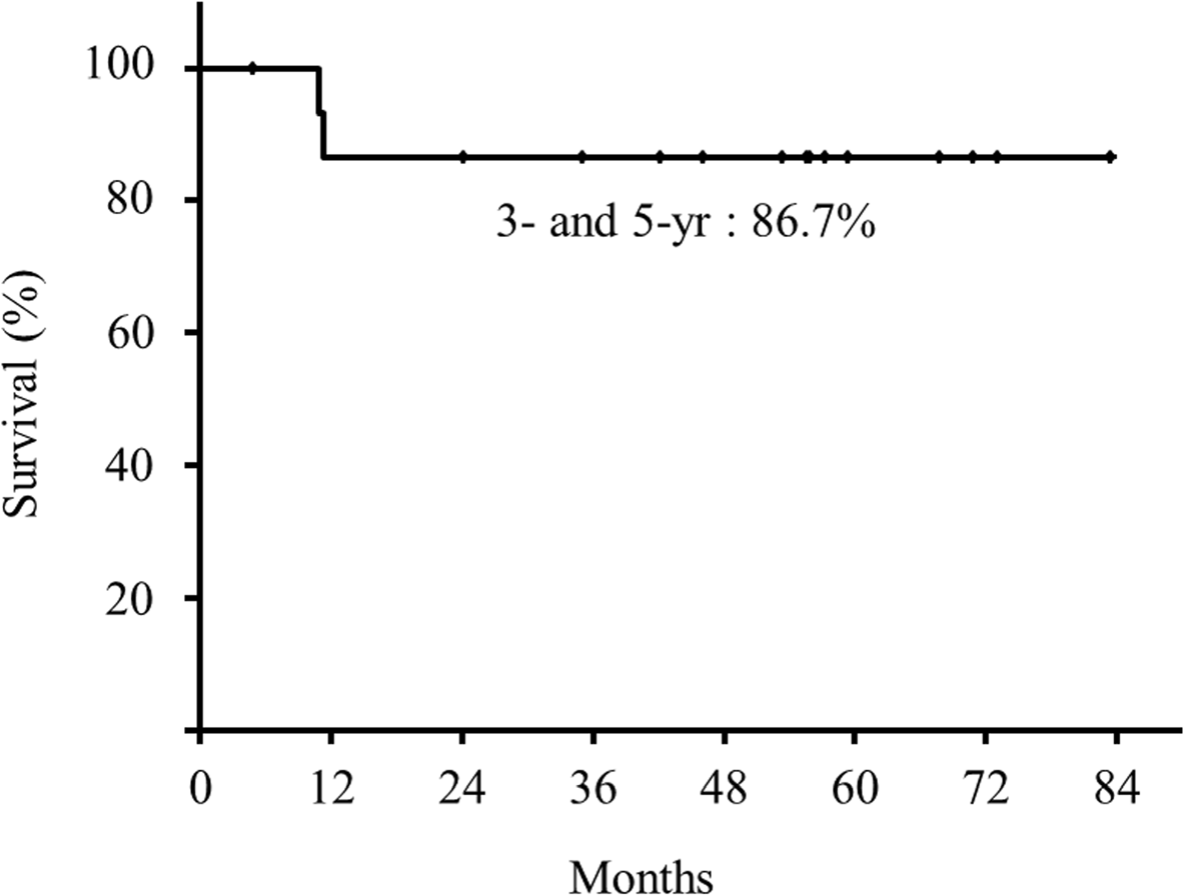
**Disease-specific survival rate.**

**Figure 5:**
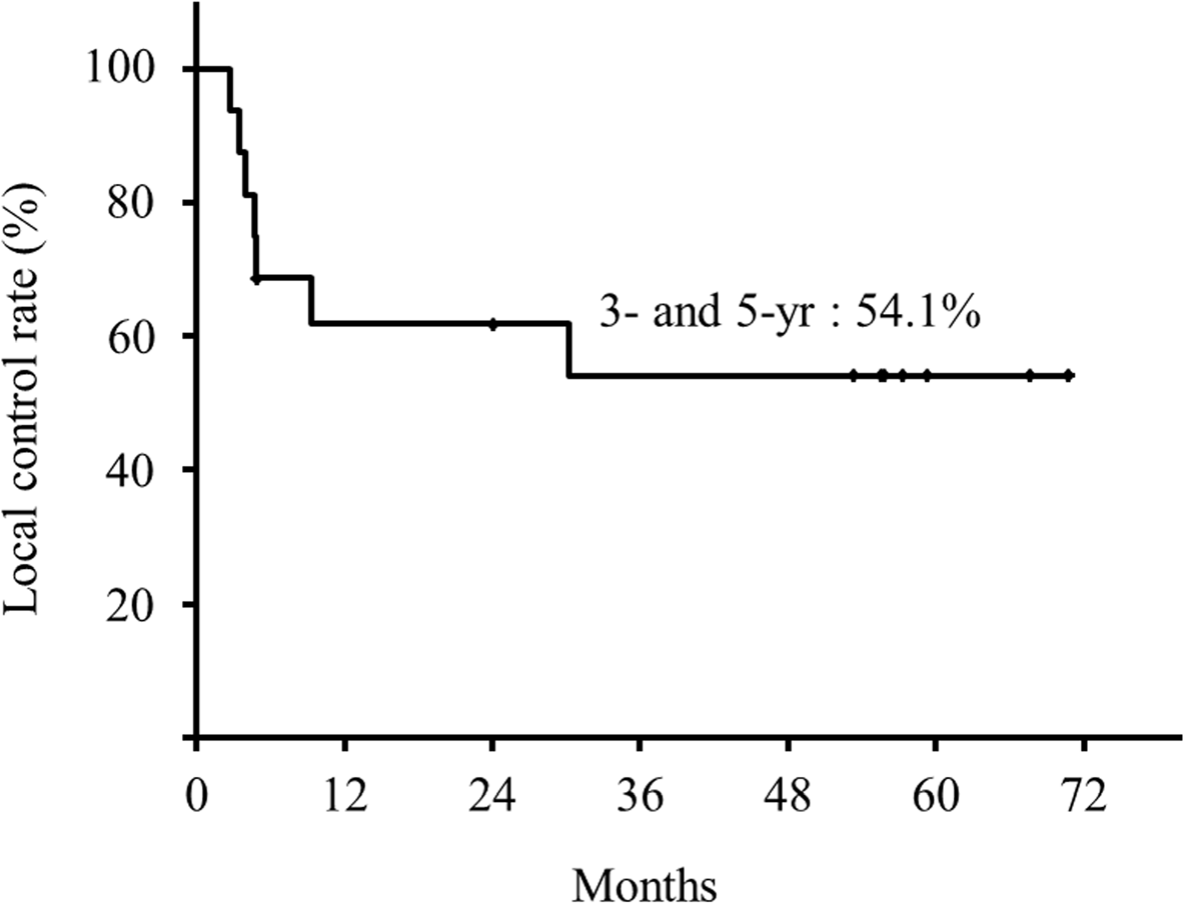
**Local control rate.**

The median follow-up period was 53.4 (range, 4.8–83.4) months. The overall 3- and 5-year survival rates were both 68.8% (Figure 3), the 3- and 5-year DSS rates were both 86.7% (Figure 4), and the 3- and 5-year local control rates were both 54.1% (Figure 5).

Seven patients (CR, four patients; PR, three patients) developed local recurrence at a median time of 3.4 (range, 1.7–29.1) months after treatment. Of these seven patients, four (CR, two patients; PR, two patients) underwent successful salvage surgery. One patient who achieved a PR and underwent SRT died of a recurrent tumor at 9.8 months after the initial treatment. In addition, one of the patients who achieved a CR but rejected additional treatment died of a recurrent tumor at 10.0 months after treatment. The disease in the other patient who achieved a CR was controlled with additional HDR brachytherapy (24 Gy in 4 fractions over 2 weeks) at 8 months after treatment. Nodal metastases occurred in one patient at 5.0 months after treatment. This patient who achieved a CR underwent successful radical neck dissection. Lung metastasis occurred in the patient who achieved a PR and underwent SRT. In two patients, local recurrence occurred at the PTV margin. Both patients had tumors in the buccal mucosa. The longest diameter of one of the tumors was 25 mm, and that of the other was 10 mm.

At the end of the follow-up period, two patients had died of the primary disease, and four patients had died of other causes (two developed pneumonia, one developed oropharyngeal cancer, and one developed esophageal cancer).

In terms of late complications, fistula formation between the skin and buccal mucosa (grade 3) was observed in the patient who underwent SRT for the primary tumor. In addition, one patient with hard palate cancer developed ulceration (grade 3) in the palatal mucosa.

## Discussion

The outcomes of LDR brachytherapy for early lip and oral cavity cancer are reportedly equivalent to those achieved using surgical treatment [1,2,13]. The effectiveness of HDR interstitial brachytherapy using the plastic tube technique for these cancers has also been established [1,2,4,5]. Some authors (including us) have reported the outcomes of HDR brachytherapy using the mold technique for early lip and oral cavity cancer, but these studies only involved a few cases with short follow-up periods [7-12]. In this retrospective study, we evaluated the outcomes of 17 patients with lip and oral cavity cancer treated using HDR brachytherapy using the mold technique, although it should be noted that their primary tumor sites varied. Our overall 5-year survival rate was 68.8%, which is not satisfactory. On the other hand, our 5-year disease-specific survival (DSS) rate was 86.7%. We consider that the difference between the overall survival and DSS rates was due to the high percentage of elderly patients (median age, 80 years) in our study. Indeed, 4 of the 17 patients (23.5%) died of other causes. One reason for the high percentage (70.6%) of elderly patients (aged > 75 years) in our study was that surgical treatment and LDR brachytherapy were avoided by the subjects, as well as by their doctors and relatives, because of the age-related decline in their performance status and physical condition. In a previous study, Khalilur et al. [3] examined patients with tongue cancer who had an age distribution similar to that in our study population. Their patients were treated with LDR brachytherapy using Au-198, and the 5-year overall survival and DSS rates were 49% and 68%, respectively [3]. Our 5-year overall survival and DSS rates were similar. However, our 5-year local control rate (54.1%) was lower than theirs (86%) [3]. In our study, local recurrence occurred in seven patients (41.2%), including three patients who achieved a PR. Three of the four patients who achieved a CR developed local recurrence within 12 months of treatment; local recurrence developed after 29.1 months in the remaining patient. As to the reason for our unsatisfactory outcomes regarding PR and local failure, we suspect that our treatment protocol might have been inappropriate.

With regard to whether or not HDR brachytherapy should be combined with EBRT, several authors have reported HDR brachytherapy protocols involving the mold technique for the treatment of oral cancer (Table 2) [7-11]. In the present study, we performed EBRT to decrease the thickness of the tumor as much as possible before the administration of HDR brachytherapy using the mold technique, and determined the total radiation dose of EBRT to be 30 Gy; this is less than the tolerated dose (TD5/5 = 32 Gy for parotid glands) of the salivary glands which are often included in the irradiation field. In the oral mucosal carcinoma, the area of radiation tumoritis, which is the early tumor reaction, was often larger than the area occupied by the gross tumor before EBRT. In those cases, we determined the GTV as the area displaying mucositis (tumoritis) after irradiation to avoid tumor recurrence in the margins.

**Table 2:** **HDR brachytherapy using mold technique for oral cavity cancers**

**Author (year)**	**Ref**	**Age**	**Sex**	**Site**	**EBRT**	**HDR brachytherapy**	**Status (months)**
Ariji (1999)	7	64	M	FM	22 Gy/11 fr	30 Gy/10 fr/5 days BID	NED (26)
		64	M	BM	26 Gy/13 fr	35 Gy/10 fr/5 days BID	NED (16)
		59	M	FM	30 Gy/15 fr	30 Gy/10 fr/5 days BID	NED (18)
		48	M	LG	40 Gy/20 fr	25 Gy/10 fr/5 days BID	NED (14)
Cengiz (1999)	8	70	F	UG	-	40 Gy/10 fr/5 days BID	NED (6)
		86	F	UG	-	40 Gy/10 fr/5 days BID	DOC (6)
Obinata (2007)	9	73	M	LG	60 Gy/24 fr	12 Gy/2 fr/2 days	Local Failur (3.5)
		76	M	UG	30 Gy/12 fr	30 Gy/5 fr/3 days	NED (18)
Garrán (2008)	10	64	F	UG	46 Gy/ 23fr	32 Gy/8 fr/4 days BID	Local Failur (7)
Kudoh (2010)	11	80	F	UG	60 Gy/30 fr	50 Gy/10 fr/5 days BID	NED (2)
		79	F	UG	60 Gy/30 fr	30 Gy/10 fr/5 days BID	NED (8)

Ref: reference, UG; upper gingiva, FM: floor of mouth, BM: buccal mucosa, LG: lower gingiva.

BID: twice a day, NED: no evidence the disease, DOC: death of cancer.

In the present study, two patients with buccal carcinoma suffered local recurrence at the PTV margin. We defined the PTV as the GTV (=CTV) + 5 mm to reduce the area at risk of complications. However, we should have considered reproducible error at every irradiation in mobile sites such as the buccal mucosa and lip. Thus, we had to add a set-up margin of about 5 mm to our PTV to prevent tumor recurrence in the margins; in contrast, this approach was not necessary for the gingiva and hard palate, which are static sites. Thus, the PTV for the tumors located in the buccal mucosa and lip might need to be defined as the GTV + 5 mm + 5 mm (set-up margin).

For HDR brachytherapy using the mold technique, the American Brachytherapy Society reported that the lips, buccal mucosa, and hard palate are suitable sites [1]. Some authors have also used this technique to treat superficial cancers of the gingiva and floor of the mouth [7-11]. In present study, regarding local recurrence at the sites of the primary tumors, cases involving the floor of the mouth (2/2) and the posterior region of the buccal mucosa (2/3) showed a high recurrence rate. In those sites, we considered that it may sometimes be difficult to hold the mold in place because of a patient’s body motion, caused by factors such as swallowing. If the mold was detached from the mucosal surface during the irradiation, the radiation dose delivered to the tumor using HDR brachytherapy might be reduced to a suboptimal level as a consequence of a change in the reference point. We suspected that floor of the mouth and the posterior region of the buccal mucosa are unsuitable sites for HDR brachytherapy using the mold technique. Indeed, when cases involving these sites were excluded, the 5-year local control rate was improved from 54.1% (n = 17) to 71.6% (n = 12). Thus, we considered that the suitable sites for HDR brachytherapy using the mold technique are the lips, anterior region of the buccal mucosa, hard palate, and gingiva.

For oral cancer, previous studies have adopted HDR brachytherapy protocols involving a range of total doses, fraction sizes, and other factors [7-11]. The American Brachytherapy Society indicated that morbidity in the oral cavity is associated with a fraction sizes > 6 Gy [1]. In the present study, we administered the prescribed HDR brachytherapy dose, which was 24 Gy delivered in four fractions, to all patients on a two fraction per day basis over a 1-week period. In our protocol, the biological effective dose (BED) delivered to the tumor at the HDR brachytherapy reference point was 38.4 Gy (32 Gy in 2 Gy equivalent fractions) according to the linear quadrant model using an α/β value of 10. In the protocols of 30 Gy in 5 fractions over 3 weeks and 36 Gy in 6 fractions over 3 weeks, the BED_10_ of them would be 48 Gy (40 Gy in 2 Gy equivalent fractions) and 57.6 Gy (48 Gy in 2 Gy equivalent fractions), respectively. On the other hand, using an α/β value of 3, the BED_3_ required to induce late normal tissue damage in these HDR brachytherapy protocols would be 90 Gy (54 Gy in 2 Gy equivalent fractions) and 108 Gy (about 66 Gy in 2 Gy equivalent fractions), respectively. Considering the BED of our HDR brachytherapy scheme, we might be able to increase the total dose of HDR brachytherapy to 30 Gy delivered in 5 fractions or 36 Gy delivered in 6 fractions to improve local control rates. However, considering severe late complication such as osteoradionecrosis, our HDR brachytherapy protocol involving the delivery of 24 Gy in 4 fractions is suitable for cancers adjacent to the jawbone, such as those in the gingiva or hard palate. On the other hand, we believe that the risk of osteoradionecrosis in cases involving tumors in the buccal mucosa and lip which are distant from the jawbone is lower than is the case for the hard palate and gingiva, although the threshold dose is not clear. Therefore, we could increase the total dose of HDR brachytherapy for tumor treatment at these sites.

## Conclusions

The outcomes of our study were by no means satisfactory when compared with those of LDR brachytherapy for lip and oral cavity cancer. Although we need to improve the technical aspects of our protocol, and carefully consider the indications for treatment, this technique is a minimally invasive treatment for lip and oral cavity cancer. Therefore, we consider that it might be an acceptable option for patients with superficial lip or oral cavity cancer, excluding some sites (floor of the mouth and posterior region of the buccal mucosa), in patients who are not eligible for aggressive treatment because of their age, performance status, or other reasons.

## Abbreviations

HDR: High-dose-rate
LDR: Low-dose-rate
EBRT: External beam radiotherapy
GTV: Gross tumor volume
CTV: Clinical target volume
PTV: Planning target volume
DSS: Disease-specific survival
CR: Complete response
PR: Partial response
SRT: Stereotactic radiotherapy
TD: Tolerated dose
BED: Biological effective dose
